# Supplementary material for: Complexity of active medicinal ingredients in radix scutellariae with sodium hydrosulfite exposure
Source: PLoS One. 2020 Sep 21;15(9):e0238927. doi: 10.1371/journal.pone.0238927 (PMC7505437; doi:10.1371/journal.pone.0238927)
Supplement: Attachment — Submitted filename: Reviewer comment_PONE-D-20-06648.docx [file pone.0238927.s001.docx]

**PLOS ONE**

**Complexity of Active Medicinical Ingredients in Radix Scutelariae with Sodium Hydrosulfite Exposure**

**Xiangcai Meng^1^, Ying Shen^1^, Wei Cong^1,2^, Ai-hua Zhang^1^, Xijun Wang^1*^**

^1^Department of Pharmacognosy, Heilongjiang University of Chinese Medicine, b4 Heping Road, Harbin 150040, China;

^2^GAP Research Center, Heilongjiang University of Chinese Medicine, Heping Road, Harbin 150040, China.

Corresponding author – Tel: +86-451-87266802. Email: 13845044491@163.com

**Reviewer comments**

The manuscript entitles “Complexity of Active Medicinical Ingredients in Radix Scutelariae with Sodium Hydrosulfite Exposure” was thoroughly reviewed to evaluate its possible acceptance in PLOS One. Overall, the results and discussion of this study are still weak. The manuscript is poorly prepared and required significant improvement. Therefore, unfortunately, this manuscript may not be suitable for publication in PLOS One.

In order to improve the quality of the manuscript, further specific comments are given below, although the list is not exhaustive:

1. First of all, it is very confusing between *Scutellaria baicalensis* and Radix Scutelariae as the author used in the title and through the text. At least, the authors should be mention Radix Scutelariae is the root of *Scutellaria baicalensis*, and used one name in all the text.
2. In the abstract and introduction part, the author mention: “The reason why plants contain so many secondary metabolites is unknown” is not exactly. Although we can not discover all the reason, numerous reason for plants contains many secondary metabolites were observed, such as:

- Some of these chemicals enhance their own survival.
- Some plants produce chemicals that act as herbicides to inhibit the growth of competing plants.
- Other plants produce substances that deter browsing by insects and herbivores
- Many plants produced secondary metabolites to respond to environmental stress (biotic, abiotic stress), etc.

3. It is not necessary and too long for explaining bout ROS and antioxidant enzyme in the introduction part. Better to focus on the main content of these results. The role of Na_2_S_2_O_4_ should be deeply mentioned.
4. In material and method: “… 2-year-old plants of *S. baicalensis* were sprayed with aqueous Na_2_S_2_O_4_ aqueous solutions at concentrations of 40 μmol/L, 0.4 μmol/L, and 0.004 μmol/L, and the control group was kept moist with just water. The samples were collected 0, 1, 2, and 3 days after spraying”. However, the result was not mentioned about the difference of result among those treatments.
5. Which treatment was showen in Table 1?
6. Result and discussion too short to difficult to understand this study.
7. The authors should establish a metabolic synthesis pathway for this plant. It helps explain the result easily.
8. There have no Fig 5L in Fig. 5.
9. All the species name in the references list should be in *Italic*.
