# Supplementary material for: Complexity of active medicinal ingredients in radix scutellariae with sodium hydrosulfite exposure
Source: PLoS One. 2020 Sep 21;15(9):e0238927. doi: 10.1371/journal.pone.0238927 (PMC7505437; doi:10.1371/journal.pone.0238927)
Supplement: Attachment — Submitted filename: Response to Reviewers.doc [file pone.0238927.s002.doc]

**Review Comments to the Author**

Please use the space provided to explain your answers to the questions above. You may also include additional comments for the author, including concerns about dual publication, research ethics, or publication ethics. (Please upload your review as an attachment if it exceeds 20,000 characters)

**Reviewer #1:** The manuscript entitles “Complexity of Active Medicinical Ingredients in Radix Scutelariae with Sodium Hydrosulfite Exposure” was thoroughly reviewed to evaluate its possible acceptance in PLOS One. Overall, the results and discussion of this study are still weak. The manuscript is poorly prepared and required significant improvement. Therefore, unfortunately, this manuscript may not be suitable for publication in PLOS One.

**Answer: Thank you for your suggestion. This manuscript aim to clarify why medicine plants contain so many active medicinical ingredients in the perspective of ecological stress. The discussion part were supplemented and throughly revised.**
In order to improve the quality of the manuscript, further specific comments are given below, although the list is not exhaustive:
1. First of all, it is very confusing between Scutellaria baicalensis and Radix Scutelariae as the author used in the title and through the text. At least, the authors should be mention Radix Scutelariae is the root of Scutellaria baicalensis, and used one name in all the text.

**Answer: Radix Scutelariae is the root of Scutellaria baicalensis, to avoid confusing, the difference between them was labeled at starting part.**

2. In the abstract and introduction part, the author mention: “The reason why plants contain so many secondary metabolites is unknown” is not exactly. Although we can not discover all the reason, numerous reason for plants contains many secondary metabolites were observed, such as:
- Some of these chemicals enhance their own survival.
- Some plants produce chemicals that act as herbicides to inhibit the growth of competing plants.
- Other plants produce substances that deter browsing by insects and herbivores
- Many plants produced secondary metabolites to respond to environmental stress (biotic, abiotic stress), etc.

**Answer: It is a problem coming from confussing expression! We mean to express the reason that plants contain so many secondary metabolites, can a few kinds of secondary metabolites with similiar effects do that? Based on this hypothesis, complexity of Active Medicinical Ingredients in Radix Scutelariae can be pesented. The manuscript was revised in another words.**

3. It is not necessary and too long for explaining bout ROS and antioxidant enzyme in the introduction part. Better to focus on the main content of these results. The role of Na2S2O4 should be deeply mentioned.

**Answer: This article aim to expound the complexity of active medicinical ingredients in Radix Scutelariae, nevertheless, active medicinical ingredients are often closely linked to the ecological stress, to which ROS and antioxidant enzymes can adjust automatically. It is probably caused by poorly expression, have been revised in article.**

4. In material and method: “… 2-year-old plants of S. baicalensis were sprayed with aqueous Na2S2O4 aqueous solutions at concentrations of 40 μmol/L, 0.4 μmol/L, and 0.004 μmol/L, and the control group was kept moist with just water. The samples were collected 0, 1, 2, and 3 days after spraying”. However, the result was not mentioned about the difference of result among those treatments.

**Answer: With aqueous Na_2_S_2_O_4_ at different concentrations, Citric acid presented a downtrend, the other ingredient a uptrend, as shown in Fig. 3.**

5. Which treatment was showen in Table 1?

**Answer: The table. 1 was all the identified components in different treatments, which has been further clarificated.**

6. Result and discussion too short to difficult to understand this study.

**Answer: A pertinent suggestion! With aqueous Na_2_S_2_O_4_ at different concentrations, The change trends has been added in Result part. The pathway of biosynthesis and transformation of secondary metabolites has been supplemented.**

7. The authors should establish a metabolic synthesis pathway for this plant. It helps explain the result easily.

**Answer: Good idea! The pathway of biosynthesis and transformation of secondary metabolites has been supplemented.**

8. There have no Fig 5L in Fig. 5.

**Answer: it is mistaken written. It has been revised.**

9. All the species name in the references list should be in Italic.
 **Answer: It has been revised.**

**Reviewer #2:** manuscript can be accepted after thorough revision keeping in view following points:

1. English writing.

**Answer: The manuscript has polished by *English Editing Services* nominated by PLOS ONE, as shown in attachment. If need to further improved, we will contact them.**

2. cite relevant references.

**Answer: It has been seriously revised according to PLOS ONE.**

3. provide some possible mechanisms in the discussion.

**Answer:	The discussion part was supplemented many information and throughly revised. The pathway of biosynthesis and transformation of secondary metabolites has been supplemented, and further provided some possible mechanisms.**

4. please rewrite conclusion and make abstract crispy and to the point.

**Answer: It is a good suggestion and has been revised.**
5. a clear hypothesis is missing.
 **Answer: It has been supplemented at last part in Introduction.**

**Reviewer #3:** Comments
1. In the Abstract of the article, line 8, it is better for the authors to add the Scutellaria inside the parentheses after the root of Scutellaria, and use it later in the whole article.

**Answer: Good idea, it has been supplemented.**

2. The authors used the Na2SO4 compound to induce oxidative treatment. What is the reason for using this compound? Explain in the article

**Answer: It has been supplemented at last part in Introduction.**

3. “Sodium Dithionite-Enhanced Quality of Scutellaria Through Modification of Secondary Metabolism” in the introduction, the last paragraph, the reference should be given.

**Answer: It is a good suggestion. It has been supplemented.**

4. In the materials section, ethanol solvents are used for extraction, while methanol solvents are commonly used to extract flavonoids. What is the reason for use? Explain the reason in manuscript

**Answer: The methanol solvents is right, the ethyl alcohol was mistakenly written.**

Results
5. In Table 1, the acronym” m / z “ should be written in Italic

**Answer: All acronym “m / z” in muanuscript has been revised into “*m / z*” .**

6. In the table in the column related to MS/MS fragment ion, for the compounds that have been identified in both positive and negative ionization methods, the negative ionization pattern is also should be written.

**Answer: Thank you for your suggestion, we have revised it in the manuscript.**

7. In Table 4, combinations 4 and 5 both have the same negative and positive ionization modes, the same m / z, and the same ionization pattern have considered while they did not use any standard combinations.

**Answer: The combinations 4 and 5 both have the same negative and positive ionization modes, the same m / z, and the same ionization pattern, they must be isomers. It is reqired standard combinations to identify them, but we have no way to obtain them, so, in article it was revised into “Aspenin-6-C-Arabinose-8-C-glucoside or Chrysin- 6-C- glucose - 8 - C - Araboside”.**

8. In Table 1, the authors include 25 compounds. Are these compounds first identified from this plant? If not, it is best to briefly explain the composition of each compound and add the relevant references.

**Answer: These compounds first identified are from *Scutellaria baicalensis*, the relevant references has been add.**

9. In Figure 2, the chromatogram images please show the root extract in the positive and negative ionization state of the peak corresponding to each combination with numbers.
 **Answer: Thank you for your suggestion, we have revised it in the manuscript.**

Discussion

10. According to statistical analysis, compounds whose VIP is larger or equal to 2 (VIP ≥2) have been selected for further study and the number of these compounds is 13, while in the discussion section of the first paragraph, 12 compounds are mistakenly written. That needs to be edited.

**Answer: It was mistaken, has been revised.**

11. In the discussion section of the last paragraph, it is better for the authors to give a brief explanation about the path of biosynthesis of flavonoids in the roots of Scutellaria.

**Answer: It is a good suggestion, has been supplemented.**

12. In the last paragraph of the last line, in explaining Figure 5, we talk about the multiplicity of biosynthetic pathways of flavonoid compounds, while Figure 5 does not. Figure 5 provides information on the content of compounds identified under different doses of oxidative treatment, and no such connection is made.

**Answer: It has been revised.**

13. In Figure 5, it is best to add a chart of the contents of the studied compounds to the image in control mode. The values provided are for treatment only

**Answer: The values have been provided in Fig.3.**
