# Supplementary material for: Complexity of active medicinal ingredients in radix scutellariae with sodium hydrosulfite exposure
Source: PLoS One. 2020 Sep 21;15(9):e0238927. doi: 10.1371/journal.pone.0238927 (PMC7505437; doi:10.1371/journal.pone.0238927)
Supplement: Attachment — Submitted filename: Response to Reviewers.doc [file pone.0238927.s003.doc]

**Response to Reviewers**

**Reviewer:** Abstract lacks flow. There is no logical connection among sentences. In needs to be rewritten.The whole text contains vernacular expessions and phrases which cannot be accepted to be published in a scientific journal. Therefore, I strongly encourage the authors to ask a senior scientist to read the manuscript and unbiasedly suggest corrections.

**Answer:** Thank you for your suggestion. The fluidity and logical relationship of the abstract in the manuscript have been revised, and AJE experts have been invited to revise the nonstandard sentences in the whole manuscript, Editing Certificate is included in the attachment. Thank you.
