# Supplementary material for: Complexity of active medicinal ingredients in radix scutellariae with sodium hydrosulfite exposure
Source: PLoS One. 2020 Sep 21;15(9):e0238927. doi: 10.1371/journal.pone.0238927 (PMC7505437; doi:10.1371/journal.pone.0238927)
Supplement: Attachment — Submitted filename: Response to Reviewers.doc [file pone.0238927.s004.doc]

**Response to Reviewers**

**Reviewer:** The authors are advised to meticulously check the text again in order to rectify vague and non-scientific expressions such as "exposed to either Na2S2O4 or suitable conditions" (Abstract).

Moreover, the text still contains sentences which lack scientific sense such as "...the most significantly different chemical markers of Na2S2O4 were shikimic acid, citric acid..." (Abstract) - I can hardly believe that sodium hidrosulfite can have chemical markers.

**Answer:** Thank you for your suggestion. There are indeed vague and non-scientific expressions in the abstract section of the manuscript. We have revised them. Thank you again for your suggestions.
